# Oral Administration of 4-Hydroxy-3-Methoxycinnamaldehyde Attenuates Atopic Dermatitis by Inhibiting T Cell and Keratinocyte Activation

**DOI:** 10.1371/journal.pone.0144521

**Published:** 2015-12-10

**Authors:** Hyun-Su Lee, Eun-Ju Choi, Heeri Choi, Kyung-Sik Lee, Hye-Ran Kim, Bo-Ra Na, Min-Sung Kwon, Gil-Saeng Jeong, Hyun Gyu Choi, Eun Young Choi, Chang-Duk Jun

**Affiliations:** 1 School of Life Sciences, Immune Synapse Research Center and Cell Dynamics Research Center, Gwangju Institute of Science and Technology, Gwangju, Republic of Korea; 2 Division of Sport Science, College of Natural Sciences, Konkuk University, Chungju, Republic of Korea; 3 College of Pharmacy, Keimyung University, Daegu, Republic of Korea; 4 College of Pharmacy, Yeungnam University, Gyeongsan, Republic of Korea; 5 Department of Biomedical Sciences, University of Ulsan College of Medicine, Seoul, Republic of Korea; Hanyang University, REPUBLIC OF KOREA

## Abstract

Atopic dermatitis (AD) is a skin condition caused by an imbalance of distinct subsets of T helper cells. Previously, we showed that 4-hydroxy-3-methoxycinnamaldehyde (4H3MC) inhibits T cell activation but does not induce apoptosis. Here, we examined the mechanism underlying the inhibitory effect of 4H3MC on AD both *in vivo* and *in vitro*. We sought to test the pharmacological effects of 4H3MC using a mouse model of 2, 4-‘2,4-dinitrocholorobenzene’ (DNCB)- and mite-induced AD. Also, we determined whether 4H3MC affects T cell differentiation and proliferation. Oral administration of 4H3MC attenuated the symptoms of DNCB- and mite-induced AD, including increased ear thickness, serum IgE levels, immune cell infiltration into inflammatory lesions, and pathogenic cytokine expression in ear tissues. *In vitro*, 4H3MC blocked T cell differentiation into Th1 and Th2 subtypes, as reflected by suppression of T-bet and GATA3, which are key transcription factors involved in T cell differentiation. In addition, 4H3MC downregulated T cell proliferation during Th1 and Th2 differentiation and keratinocyte activation. Collectively, these findings suggest that 4H3MC ameliorates AD symptoms by modulating the functions of effector T cells and keratinocytes.

## Introduction

Atopic dermatitis (AD) is a chronic skin disease resulting from both an imbalanced immune response and genetic and environmental factors [1]. Because many people worldwide suffer from AD, much research has been carried out to develop fundamental therapies that are safe to administer and exhibit fewer side effects. Hundreds of potential molecules and agents and their mechanisms of action have been introduced; however, few molecules have clinical utility for economic and safety reasons.

Once dendritic cells (DCs) in the draining lymph nodes (dLNs) present an antigen to naïve Th0 cells, the latter are primed to differentiate into Th2 effector cells if this occurs in the presence of IL-4 secreted by activated Th2 cells and TSLP (Thymic stromal lymphopoietin) secreted by activated keratinocytes. Acute AD develops within 24 hours of allergen invasion, primarily as a result of Th2 cells and the cytokines that they secrete, which include IL-4, IL-5, IL-13, and IL-31 [2,3]. As the acute conditions become chronic, AD skin undergoes tissue remodeling due to chronic inflammation driven by Th1/Th17 effector cells. In particular, Th1 cytokines stimulate keratinocytes to produce pro-inflammatory cytokines such as TNF-α, IL-1β, and IL-6, thereby amplifying inflammation at the site of the lesion [2,4]. Thus, regulating the functions of T cells and other cells at inflammatory sites is crucial if we are to treat AD successfully.

Phenolic compounds, including gallic acid, caffeic acid [5], and ferulic acid [6] have anti-inflammatory [7], antimicrobial [8], hypolipidemic [9], and anti-mutagenic [10], and anti-carcinogenic properties [10,11]. We previously showed that the phenolic compound 4-hydroxy-3-methoxycinnamaldehyde (4H3MC) suppresses T cell activation [12]. Therefore, we speculated that 4H3MC might have therapeutic potential and alleviate the symptoms of AD. The aim of the present study was to examine whether 4H3MC regulates T cell activation and concurrent inflammation to control AD symptoms in mice.

## Materials and Methods

### Mice

Eight-week-old female BALB/c and C57BL/6 mice were purchased from Samtako (Osan, Korea) and housed in specific pathogen-free conditions. All experiments were approved by the Animal Care and Use Committee of the School of Life Sciences, Gwangju Institute of Science and Technology (approval number, GIST2014-31).

### Preparation of 4H3MC from *C*. *longa* roots

The air-dried rhizomes (6.0 kg) were extracted with MeOH (10 L) at room temperature for 5 days. The extract (1.2 kg) was suspended in water and partitioned three times with the same volume of ethyl acetate. The ethyl acetate extract (70 g) was fractionated by silica gel column chromatography and eluted with a gradient of CH_2_Cl_2_-ethyl acetate (from 10:0 to 1:1) to yield seven fractions (Fr. 1–7). Fr. 3 (4.3 g), which contained the greatest amount of the compound of interest, was purified by recrystallization from cold MeOH (yield: 482.8 mg, 0.69% (w/w)). Spectroscopic and mass spectrometry analyses and comparisons with data published in the literature identified the compound as 4H3MC [13].

### Reagents and cell culture

DNCB (2, 4-dinitrochlorobenzene), mite extract, phorbol 12-myristate 13-acetate (PMA), A23187, and carboxyfluoresceinsuccinimidyl ester (CFSE) were purchased from Sigma (St. Louis, MO). FITC-anti-mouse CD4, PerCP cy5.5-anti-mouse IFN-γ, PE-anti-mouse IL-4, and FITC-anti-mouse CD4 were obtained from e-Bioscience (San Diego, CA). A mouse IgE ELISA kit, purified rat anti-mouse IFN-γ, and purified rat anti-mouse IL-12 were obtained from BD Biosciences (San Jose, CA). Mouse anti-CD28, mouse IL-4 ELISA kit, recombinant human IFN-γ, and recombinant human TNF-α were purchased from R&D Systems (Minneapolis, MN). Recombinant mouse IL-4 was obtained from Peprotech (Hamburg, Germany). The 145-2C11 (mouse anti-CD3; CRL-1975) hybridoma cell line was purchased from the ATCC (Manassas, VA). HaCaT keratinocytes were cultured in RPMI 1640 containing 2 mM L-glutamine, antibiotics (100 μg/mL streptomycin, 100 U/mL penicillin), and 10% fetal bovine serum. Cells were incubated at 37°C in a humidified atmosphere of 5% CO_2_.

### Induction of AD

**Fig 1:**
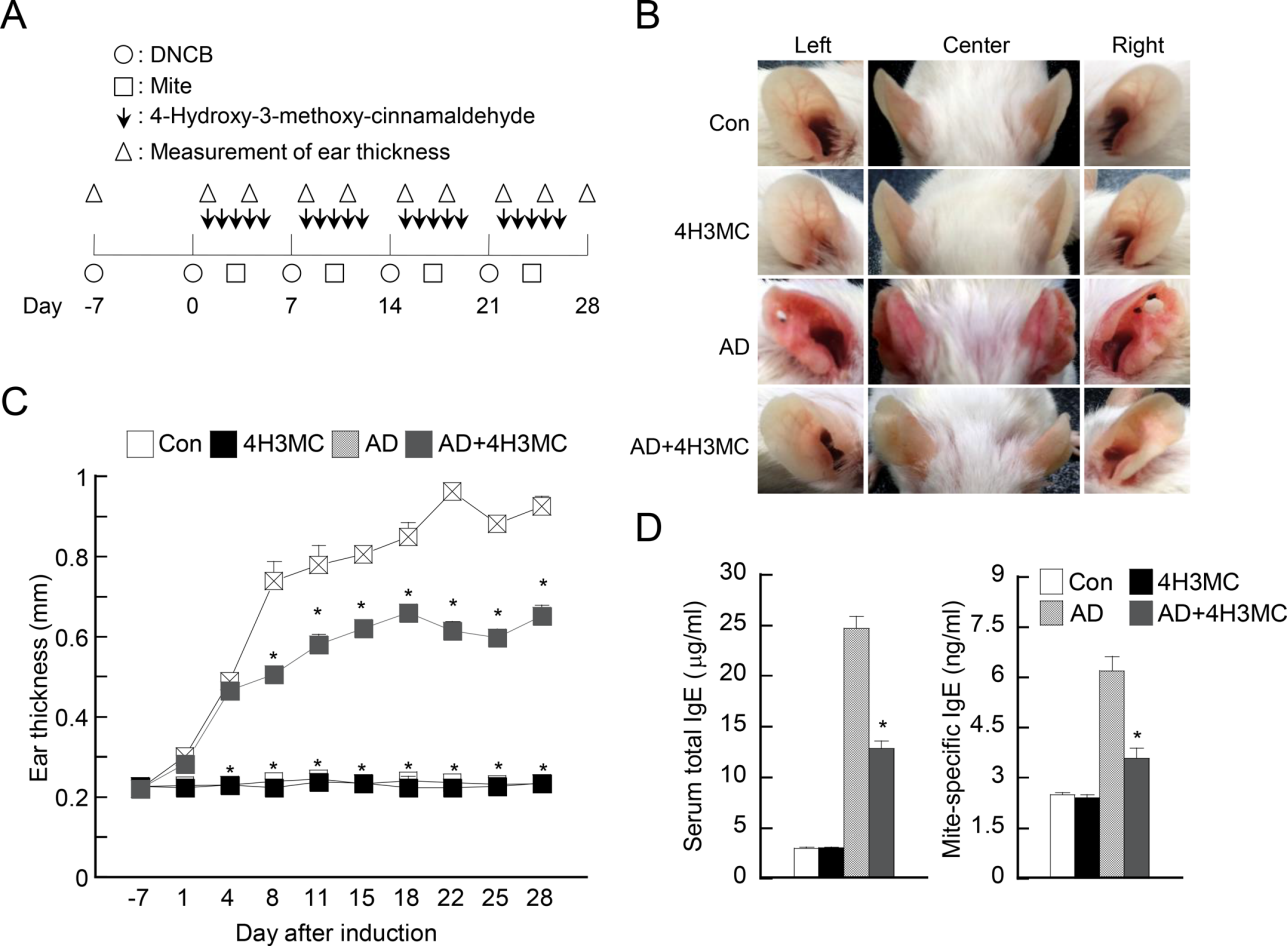
Oral delivery of 4H3MC ameliorates the symptoms of atopic dermatitis in mice. (A) A schematic diagram showing the induction and treatment of atopic dermatitis (AD). (B) Representative pictures of mouse ears on Day 28 (n = 3–6/group). Con, control mice; 4H3MC, control mice receiving 4H3MC; AD, AD mice; AD+4H3MC, AD mice receiving 4H3MC. (C) Ear thickness during the course of AD. (D) Levels of serum IgE and mite-specific IgE in mice were measured by ELISA. Blood samples were collected by cardiac puncture at Day 28 post-induction. Data are expressed as the mean ± SEM. *P < 0.05, *versus* the AD control group.

AD was induced using DNCB and mite extract, as previously described [14]. A schematic diagram of the experimental procedure is shown in Fig 1A. Briefly, BALB/c mice were divided into four groups and the surface of both earlobes was stripped five times with surgical tape (Seo-il chemistry, Hwa-sung, Korea). After stripping, 20 μL DNCB (1%) was painted onto each ear (Day 0), followed by 20 μL mite extract (10 mg/mL) on Day 4. Thereafter, DNCB and mite extract were applied alternately at 3–4 day intervals for 4 weeks. Mice received a daily dose of 4H3MC (50 mg/kg) for 4 weeks, starting at Day 1. A dial thickness gauge (Kori Seiki MFG Co., Japan) was used to measure ear thickness 24 h after the application of DNCB or mite extract. At Day 28, blood samples were collected by cardiac puncture and plasma stored at—70°C until further analysis. After blood collection, ears were excised and subjected to histopathological analysis.

### Histological analysis

Ears from each group were fixed in 10% paraformaldehyde and embedded in paraffin. Paraffin blocks were sliced into 5 μm-thick sections, deparaffinized, and stained with hematoxylin and eosin (H&E). The thickness of the epidermis and dermis on the sections was measured. To count infiltrating mast cells, sliced sections were stained with 0.01% toluidine blue and mast cells counted at five random sites. To count the number of T cells infiltrating the ear tissues, paraffinized blocks were sliced and stained with FITC-anti-mouse CD4. Fluorescence was measured under a confocal microscope and CD4^+^ T cells were counted at five random sites.

### ELISA

Differentiated Th1 and Th2 cells (1 × 10^6^/well) were seeded into a 24-well plate and pre-incubated with 4H3MC (10 μM) for 30 min. The cells were then stimulated with anti-CD3/CD28 antibodies or PMA/A23187 for 24 h. The supernatants were collected and the levels of IFN-γ and IL-4 measured using an ELISA duo set kit (R&D Systems, Minneapolis, MN).

### Measurement of serum IgE

Blood samples from each group were collected by cardiac puncture after sacrifice on Day 28. The levels of total IgE and mite-specific IgE in the sera were measured using commercial ELISA kits according to the manufacturer’s instructions.

### Real-time PCR

Total RNA was isolated from ear tissues or from CD4^+^ T cells isolated from spleen, dLNs, and non-draining lymph nodes (non-dLN) from each group using TRI Reagent (Molecular Research Center, Cincinnati, OH). RNA was reverse-transcribed using RT Premix (Enzynomics, Daejeon, Korea). PCR was then performed using the primers listed in Table 1. The amplification protocol was as follows: denaturation at 72°C for 7 min, followed by 30 cycles of denaturation at 94°C for 30 s, annealing at 60–62°C for 20 s, and extension at 72°C for 40 s. PCR amplification was performed using a StepOne Real-Time PCR System (Applied Biosystems, Waltham, MA), which allowed continuous fluorescence detection in a total volume of 10 μL cDNA/control, gene-specific primers, and SYBR Premix Ex Taq (Takara Bio Inc., Shiga, Japan). The mRNA levels of the target genes were normalized relative to *GAPDH*, using the following formula: relative mRNA expression = 2^-(ΔCt of target gene-ΔCt of GAPDH)^, where Ct is the threshold cycle value. The levels of mRNA were expressed as the relative fold change.

**Table 1:** PCR primer sequences for mRNA quantification.

mouse *TNF-α*	F	5’-aagcctgtagcccacgtcgta-3’
	R	5’-ggcaccactagttggttgtctttg-3’
mouse *IFN-γ*	F	5’-tcaagtggcatagatgtggaagaa-3’
	R	5’-tggctctgcaggattttcatg-3’
mouse *IL-4*	F	5’-acaggagaagggacgccat-3’
	R	5’-gaagccgtacagacgagctca-3’
mouse *IL-5*	F	5’-gaagtgtggcgaggagagac-3’
	R	5’-gcacagttttgtggggtttt-3’
mouse *IL-6*	F	5’-ccggagaggagacttcacag-3’
	R	5’-ggaaattggggtaggaagga-3’
mouse *IL-13*	F	5’-gcaacatcaacaggaccaga-3’
	R	5’-gtcagggaatccagggctac-3’
mouse *IL-31*	F	5’-tcggtcatcatagcacatctggag-3’
	R	5’-gcacagtccctttggagttaagtc-3’
mouse *IL-17*	F	5’-tcccctctgtcatctgggaag-3’
	R	5’-ctcgaccctgaaagtgaagg-3’
mouse *TSLP*	F	5’-aggctaccctgaaactgag-3’
	R	5’-ggagattgcatgaaggaatacc-3’
mouse *CD117*	F	5’-caaggcttctccaattctgc-3’
	R R	5’-tgcagtggtccacagaagag-3’
mouse *FCER1G*	F	5’-ctccttttggtggaacaagc-3’
	R	5’-gggtaaggacaataccatacaaaaa-3’
mouse *CCR2*	F	5’-cctgcaaagaccagaagagg-3’
	R	5’-gtgagcaggaagagcaggtc-3’
mouse *CD4*	F	5’-gagagtcagcggagttctc-3’
	R	5’-ctcacaggtcaaagtattgttg-3’
mouse *CD19*	F	5’-ctgcctggacagtgaacgtg-3’
	R	5’-acagccaaagtgtggagccg-3’
mouse *CD11c*	F	5’-acacagtgtgctccagtatga-3’
	R	5’-gcccagggatatgttcacagc-3’
mouse *T-bet*	F	5’-agcaaggacggcgaatgtt-3’
	R	5’-gggtggacatataagcggttc-3’
mouse *GATA3*	F	5’-ctcggccattcgtacatggaa-3’
	R	5’-ggatacctctgcaccgtagc-3’
mouse *GAPDH*	F	5’-gcacagtcaaggccgagaat-3’
	R	5’-gccttctccatggtggtgaa-3’
human *TNF-α*	F	5’-cctaccagaccaaggtcaac-3’
	R	5’-agggggtaataaagggattg-3’
human *IL-1β*	F	5’-ggatatggagcaacaagtgg-3’
	R	5’-atgtaccagttggggaactg-3’
human *IL-6*	F	5’-aaagaggcactgccagaaaa-3’
	R	5’-atctgaggtgcccatgctac-3’
human *TSLP*	F	5’-tagcaatcggccacattgcct-3’
	R	5’-gaagcgacgccacaatccttg-3’
human *GAPDH*	F	5’-cggagtcaacggatttggtcgtat-3’
	R	5’-agccttctccatggtggtgaagac-3’

### T cell differentiation

C57BL/6 mice were sacrificed and CD4^+^ T cells isolated from LNs and spleens by MACS cell separation (Miltenyi Biotec, Bergisch Gladbach, Germany). Isolated cells (1 × 10^6^/well) were incubated in plates pre-coated with an anti-mouse CD3 antibody (2 μg/mL) and then treated with a soluble anti-mouse CD28 antibody (2 μg/mL). Anti-mouse IL-4 antibodies (10 μg/mL), recombinant mouse IL-12 (10 ng/mL), and recombinant human IL-2 (2 IU) were added to the cell culture to induce differentiation into Th1 cells. Alternatively, anti-mouse IFN-γ (5 μg/mL), anti-mouse IL-12 (5 μg/mL), recombinant mouse IL-4 (10 ng/mL), and recombinant human IL-2 (2 IU) were added to the cultures to induce differentiation into Th2 cells. Fresh human IL-2 recombinant protein (2 IU) was added daily. After 5 days, differentiated cells were harvested and total RNA extracted to examine expression of the T-bet and GATA3 transcription factors.

### Intracellular cytokine staining

To determine differentiation efficiency, Th1- or Th2-polarized cells were cultured with 4H3MC, harvested, and seeded on a 12-well plate (1 × 10^6^/well). Cells were then stimulated with PMA/A23187 for 2 h. Brefeldin A (10 μg/mL) was added and the cells incubated for a further2 h. Cells were then collected, fixed with 4% paraformaldehyde, permeabilized with 0.1% saponin solution, and stained with anti-mouse IFN-γ or anti-mouse IL-4 antibodies. Samples were washed twice with PBS and fluorescence measured in a flow cytometer (BD, FACSCanto II). Data were analyzed using FlowJo software.

### Proliferation assay

CD4^+^ T cells (1 × 10^6^) isolated from LNs and spleen were stained with 10 μM CFSE (Molecular Probes, Carlsbad, CA) in a 37°C incubator for 30 min. CFSE-labeled cells were then cultivated with cytokines and antibodies (to induce Th1/Th2 differentiation as described above) together with 4H3MC (10 μM). After 72 hours, cells were collected and subjected to flow cytometry to analyze CFSE fluorescence intensity.

### HaCaT cell activation

For the dose-dependent experiments, human keratinocytes (HaCaT cells; 1 × 10^6^) were incubated with the indicated concentrations (0–20 μM) of 4H3MC for 30 min. The cells were then stimulated with TNF-α (10 ng/ml) and IFN-γ (10 ng/ml) for 3 h. For the time-dependent experiments, HaCaT cells (1 × 10^6^) were incubated with 10 μM 4H3MC for 30 min. The cells were then stimulated with TNF-α (10 ng/ml) and IFN-γ (10 ng/ml) for the indicated times (0–6 h).

### Protein kinase C (PKC) activity assay

PKC activity was measured as previously described [12]. Briefly, human HaCaT cells (1 × 10^6^) were suspended in lysis buffer (1% Triton X-100, 150 mM NaCl, 20 mM Tris pH 7.5, and protease and phosphatase inhibitors), kept on ice for 1 h, and then centrifuged at 14,000 g at 4°C for 30 min. The cell lysate was incubated with 4H3MC (0–20 μM) at 4°C for 30 min, followed by addition of PMA (200 nM). After incubation for 3 min, PKC activity was measured using an ELISA-based nonradioactive protein kinase assay kit, in which a synthetic peptide acted as a substrate for PKC and a polyclonal antibody recognized the phosphorylated form of the substrate. The assay was developed with tetramethylbenzidine (the color developed in proportion to PKC phosphotransferase activity). The intensity of the color was measured at 450 nm. Data were expressed as relative kinase activity.

### Statistical analysis

Data were expressed the mean of at least three independent experiments conducted on different days. Significance was tested using an unpaired Student’s *t*-test and one-way analysis of variance. P < 0.05 was considered significant.

## Results

### Oral delivery of 4H3MC ameliorates symptoms of AD in mice

We previously showed that 4H3MC inhibits T cell activation by targeting PKC and its downstream signaling pathways, but it does not trigger cell death [12]; therefore, we reasoned that 4H3MC may be used as an immunomodulator to limit AD because T cells make a significant contribution to the development and maintenance of this condition. To test this, we induced AD in BALB/c mice by applying DNCB and mite extract to the ears and then treated them by oral administration of 4H3MC (Fig 1A). We observed mice ears up until Day 28 post-application and collected blood after sacrifice. The ears of AD mice became red and swollen after 28 days. Interestingly, AD mice receiving 4H3MC showed attenuated symptoms (Fig 1B). When ear thickness over time was measured and compared between groups, we found that thickness increased as AD developed. Oral administration of 4H3MC to AD mice led to a noticeable reduction in ear thickness, which was significant (p>0.05) at Day 8 and after (Fig 1C). As IgE production is an indicator of AD development and increased levels are critical for atopic pathogenesis [15], we also measured IgE levels in AD mice. As expected, AD mice receiving 4H3MC produced significantly less IgE and mite-specific IgE than AD control mice at Day 28 (Fig 1D). These data indicate that 4H3MC ameliorates AD symptoms in mice and suggest that 4H3MC might be an effective modulator of immune responses in AD.

### Oral delivery of 4H3MC reduces tissue inflammation and infiltration of immune cells

**Fig 2:**
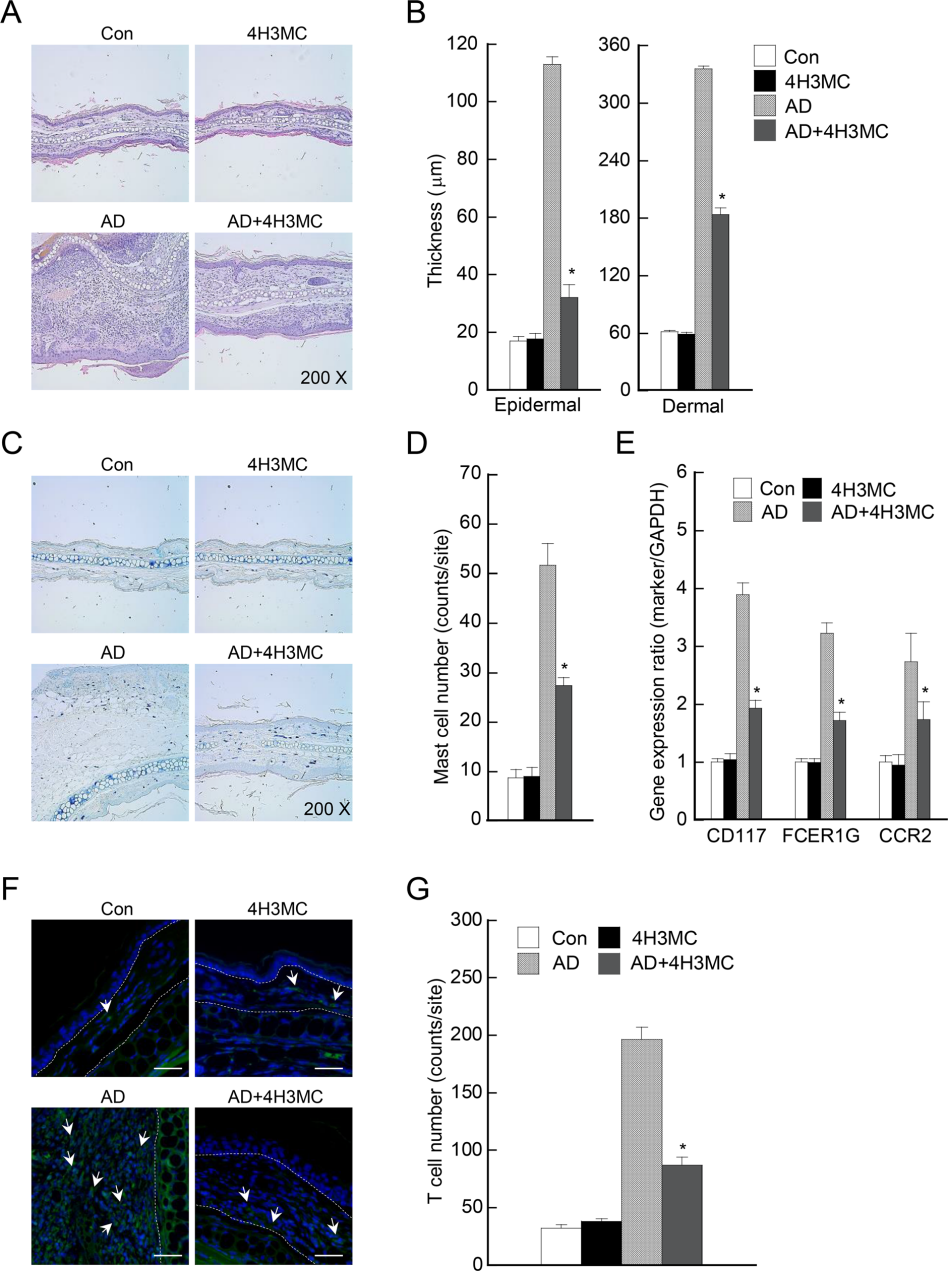
4H3MC reduces tissue inflammation and infiltration of immune cells in AD mice. Microphotographs of left ear sections isolated at Day 28 post-AD induction and stained with (A) hematoxylin and eosin (H&E), (C) toluidine blue, and (F) an anti-CD4 antibody. Original magnification, ×200. White bar, 10 μm (F). (B) Epidermal and dermal thickness was measured in H&E-stained microphotographs. (D) Infiltrating mast cells were counted after toluidine blue staining. (E) mRNA levels of mast cell markers (CD117, FCER1G and CCR2) in the ear tissues at Day 28 were analyzed by real-time PCR. (G) Infiltrating CD4^+^ T cells were counted after staining with anti-CD4 antibodies. Data are expressed as the mean ± SEM (n = 3–6/group). *P < 0.05, *versus* the AD control group.

Skin lesions associated with AD have typical microscopic characteristics, which include hyperkeratosis, parakeratosis, and acanthosis, along with infiltration of inflammatory cells. Hence, we next sought to assess skin manifestations microscopically in AD mice and AD mice receiving 4H3MC. To this end, we induced AD in mice, excised the ears, and examined the thickness of epidermal and dermal tissues and the number of mast cells and T cells in the skin lesions. First, H&E staining revealed that the ears of AD mice showed extensive epidermal and dermal changes, whereas these changes were attenuated in AD mice receiving 4H3MC (Fig 2A). The epidermal and dermal tissues in AD mice were significantly thinner after administration of 4H3MC (Fig 2B). Second, toluidine blue staining of ear tissue sections showed mast cell infiltration into the epidermis and dermis of AD mice; this was abrogated by administration of 4H3MC (Fig 2C and 2D). More specifically, we analyzed the expression of mast cell markers CD117, FcRγ and CCR2 in the ear tissues. As shown in Fig 2E, mRNA levels for mast cell markers were significantly lower after 4H3MC administration. However, there was no significant difference in cell death in the ear tissues after 4H3MC treatment, as revealed by TUNEL assay (data not shown), indicating that oral administration of 4H3MC inhibits the recruitment of mast cells, but does not induce cell death of mast cells. Third, we used a fluorescence-conjugated anti-CD4 antibody to measure T helper cell infiltration into the ear tissues. When left untreated, high numbers of CD4^+^ cells infiltrated the ear tissues of AD mice; however, there was a significant reduction after treatment with 4H3MC (Fig 2F and 2G). Taken together, these findings suggest that 4H3MC reduces inflammation and immune cell infiltration in the skin, thereby attenuating AD.

### Oral delivery of 4H3MC reduces the production of inflammatory cytokines in ear tissues

**Fig 3:**
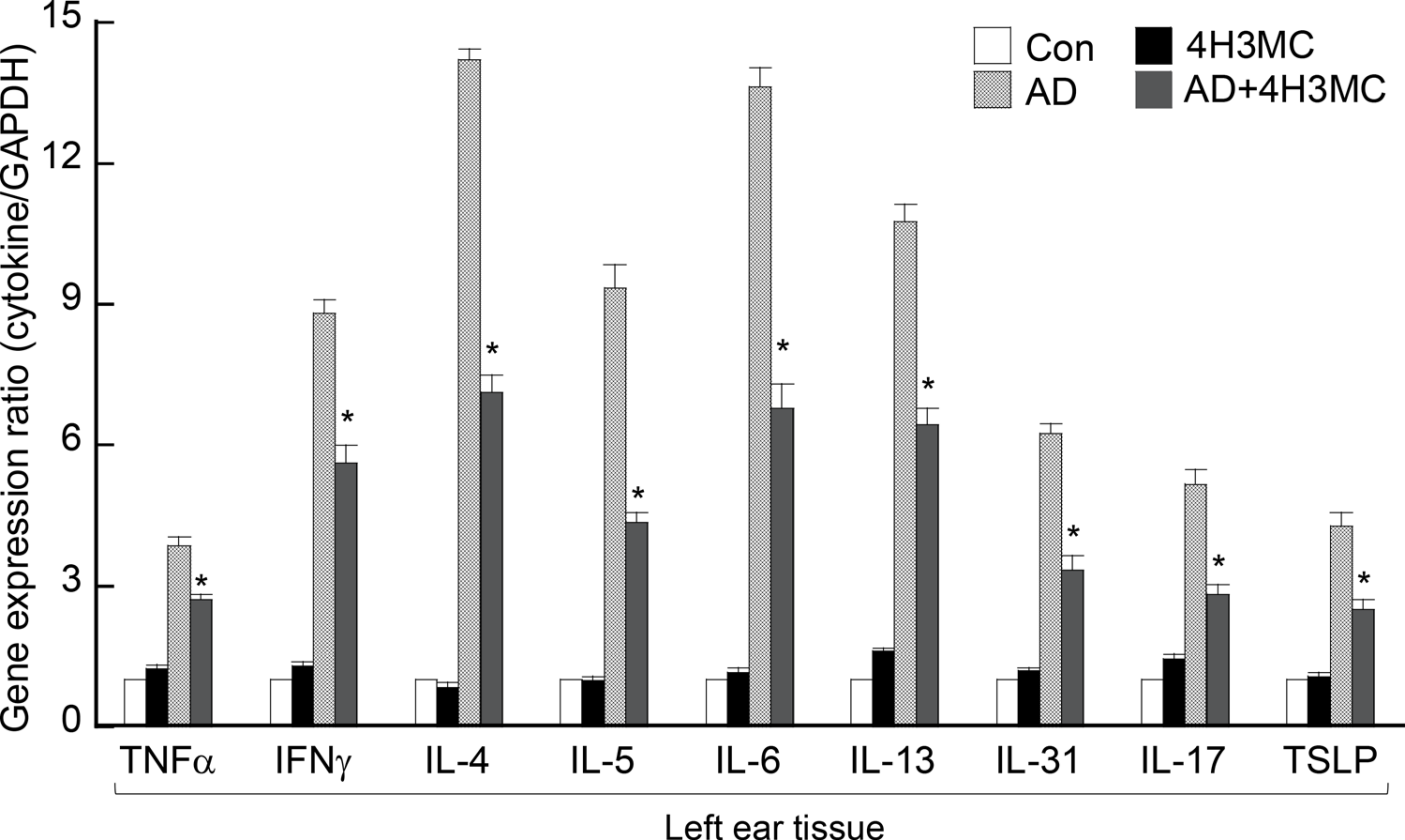
4H3MC inhibits the production of pro-inflammatory cytokines in ear tissues of AD mice. Expression of cytokines in left ear tissues from each group was measured by real-time RT-PCR. mRNA was isolated at Day 28 post-AD induction. Data are expressed as the mean ± SEM (n = 3–6/group). *P < 0.05, *versus* the AD control group.

Infiltrating T cells and T cell-derived cytokines are crucial for AD development; therefore, we next examined whether there was any difference in the levels of cytokines secreted by distinct T cell subsets, specifically cytokines derived from effector T cells such as Th1, Th2, and Th17, between the AD mice and AD mice receiving 4H3MC. At Day 28, RNA was extracted from ear tissues and then analyzed by RT-PCR. Ear tissues harvested from AD mice contained a variety of inflammatory cytokines. However, ear tissues from AD mice receiving 4H3MC contained significantly lower levels of TNF-α, IFN-γ, IL-4, IL-5, IL-6, IL-13, IL-17, and IL-31 (Fig 3). More interestingly, the reduction in the levels of Th2-type cytokines (IL-4, IL-5, IL-6, IL-13, and IL-31), which are important for AD pathogenesis, was greater than that of Th1-type cytokines [16]. Additionally, expression of a keratinocyte-derived cytokine, TSLP, was analyzed in ear tissues. Mice receiving 4H3MC showed significantly lower levels of TSLP than AD controls (Fig 3). Collectively, these data suggest that 4H3MC regulates CD4^+^ effector T cell responses and keratinocyte function.

### Oral delivery of 4H3MC controls immune responses in the spleen and lymph nodes in AD

**Fig 4:**
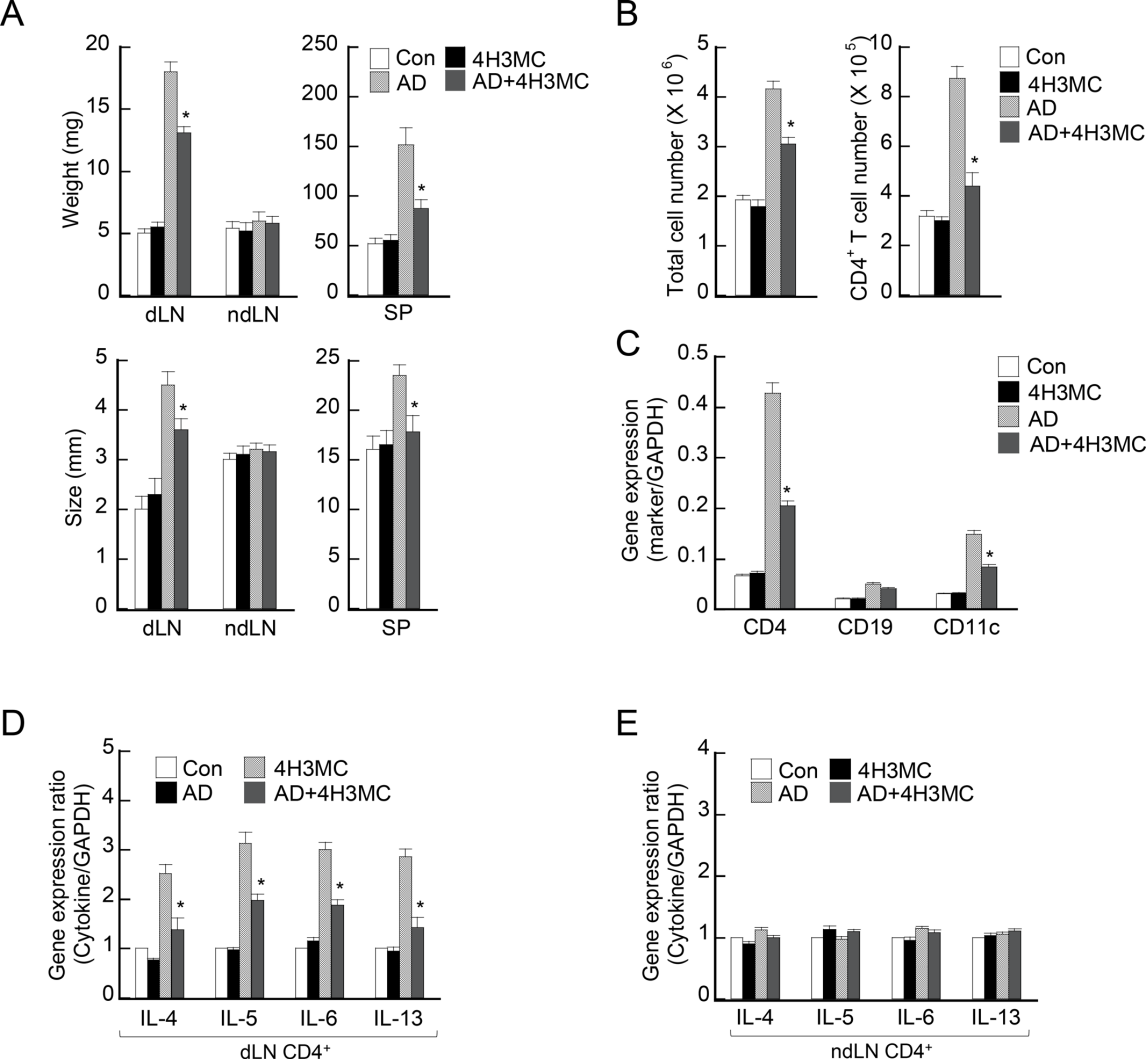
4H3MC induces morphological changes in immunological organs and down-regulates the production of pro-inflammatory cytokines in dLNs and spleens (but not in non-dLNs). (A) Weight and size of dLNs, non-dLNs, and spleens from each mouse were measured at Day 28 post-AD induction. (B) The number of total cells and CD4^+^ T cells in the dLNs was counted. (C) CD4, CD19 and CD11c mRNA in the dLNs was analyzed. (D) and (E) Expression of genes encoding pro-inflammatory cytokines in CD4^+^ T cells from (D) dLNs and (E) non-dLNs. On Day 28, dLNs and non-dLNs were removed and CD4^+^ T cells isolated by MACS. After isolation of total RNA, expression of cytokine genes was measured by real-time RT-PCR. Data are expressed as the mean ± SEM (n = 3–6/group). *P < 0.05, *versus* the AD control group.

Alteration in serum IgE levels by long-term oral delivery of 4H3MC reflects a systemic change in the immune response in AD mice. Therefore, we next examined the immunological organs of AD mice following 4H3MC administration. At Day 28 post-AD induction, we measured the weight and size of the spleen and lymph nodes. As expected, there was an increase in both the weight and size of the spleen and dLNs (but not the non-dLNs) in AD mice (Fig 4A). The increase in organ size was much smaller in AD mice receiving 4H3MC. To determine the mechanism by which the size of dLN was reduced upon treatment with 4H3MC, we analyzed cellular components in the dLN of the AD mice receiving saline or 4H3MC. Total cell number of dLNs was increased in the AD mice but 4H3MC treatment significantly blunted the increase, indicating that 4H3MC inhibits leukocyte recruitment to dLN that occurs upon AD development (Fig 4B). More specifically, the number of CD4^+^ T cells and the level of CD4 mRNA, as well as that of CD11c mRNA (an indicator of DCs), was significantly lower in the LNs of AD mice receiving 4H3MC than in AD control mice (Fig 4B and 4C). These data suggest that 4H3MC alleviates AD symptoms by regulating immune cell recruitment to the dLN. In accordance with these findings, at Day 28 the production of Th2-type cytokines by CD4^+^ T cells isolated from dLNs was significantly lower than that in control AD mice (Fig 4D). There was no difference in cytokine production by CD4^+^ cells in the non-dLNs of AD or control mice (Fig 4E). These results indicate that 4H3MC attenuates systemic immune responses in AD mice.

### 4H3MC inhibits T cells both before and after differentiation

**Fig 5:**
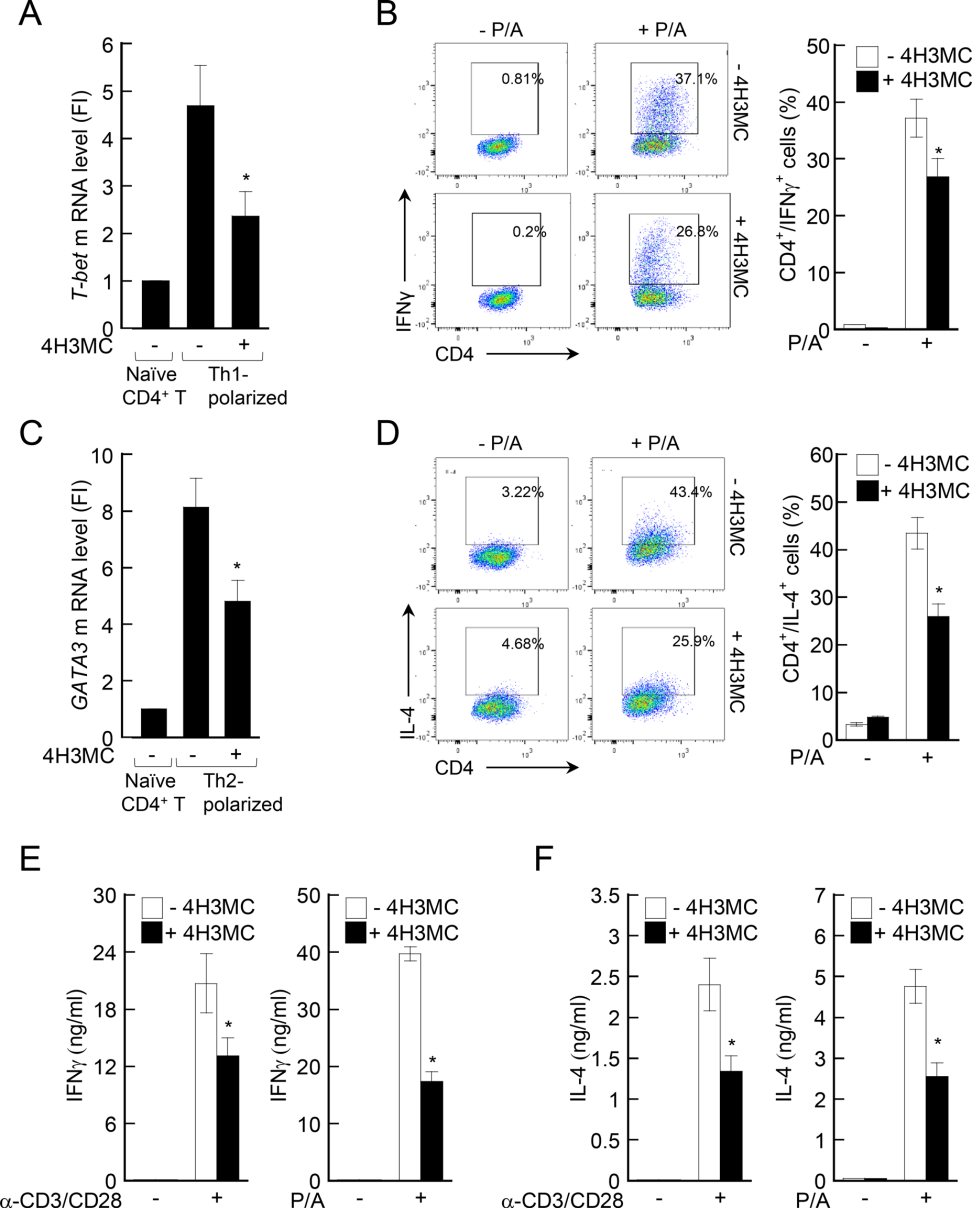
4H3MC inhibits T cells both before and after differentiation. (A–D) CD4^+^ T cells were isolated from normal C57BL/6 mice and differentiated *in vitro*, as described in Materials and Methods, in the absence or presence of 4H3MC. On Day 5 of culture (A) Th1-polarized cells or (C) Th2-polarized cells were collected and total RNA isolated. Expression of (A) T-bet and (C) GATA3 mRNA was measured by real-time RT-PCR. (B) and (D) On Day 5 of culture (B) Th1-polarized cells and (D) Th2-polarized cells were treated with PMA/A23187 for 4 h. Two hours before cell harvest, brefeldin A was added to the cultures. Production of IFN-γ (B) or IL-4 (D) was measured by FACS analysis. (E) and (F) CD4^+^ T cells were isolated from normal mice and differentiated. On Day 5 of culture, differentiated Th1 (E) cells or Th2 (F) cells (1 × 10^6^/sample) were pre-incubated with 4H3MC (10 μM) for 30 min and stimulated with anti-CD3/CD28 antibodies (left) or PMA/A23187 (right) for 24 h. The levels of IFN-γ (E) or IL-4 (F) were then measured by ELISA. Data are expressed as the mean ± SD from three independent experiments. *P < 0.05, *versus* the cells not treated with 4H3MC.

*In vivo* studies showed that 4H3MC suppresses AD symptoms. In addition, 4H3MC regulates the ability of T cells to produce cytokines, both local and systemically, as revealed in the spleens and dLNs of AD mice. Given that such cytokines produced by effector T cells, if uncontrolled, might be a fundamental cause of AD symptoms, we next examined the mechanisms by which 4H3MC regulates T cell function. To examine whether 4H3MC directly regulates T cell differentiation, we isolated naïve CD4^+^ T cells from the spleens and LNs of C57BL/6 mice and incubated them with specific combinations of cytokines and antibodies to induce Th1- and Th2-polarization *in vitro*. Concurrently, 4H3MC was added to the cultures. Fig 5A and 5C show that 4H3MC reduced the levels of T-bet and GATA3 mRNA, which are master transcription factors for Th1 or Th2 differentiation, respectively, in Th1- and Th2-polarized cells. Similar findings were observed in T cells isolated from the LNs and spleens of BALB/c mice (data not shown). In addition, 4H3MC reduced the populations of IFN-γ-producing cells (Th1-polarized cells) and IL-4-producing cells (Th2-polarized cells) within the total CD4^+^ T cell population after stimulation with PMA/A23187 (Fig 5B and 5D). This suggests that 4H3MC regulates T cell differentiation. We then tested whether 4H3MC affected cytokine production by differentiated Th1-polarized and Th2-polarized cells. To do this, we isolated naïve CD4^+^ T cells from C57BL/6 mice and induced them to differentiate, as described above. Once differentiated, the cells were pre-incubated for 30 min either with or without 4H3MC and then stimulated with anti-CD3/CD28 antibodies or PMA/A23187. The differentiated Th1-polarized cells produced IFN-γ, whereas Th2-polarzied cells produced IL-4. Pre-incubation of differentiated Th1 and Th2 cells with 4H3MC led to a significant decline in cytokine production (Fig 5E and 5F). These results suggest that 4H3MC affects T cell differentiation and subsequent cytokine production.

### 4H3MC inhibits T cell proliferation during Th1/Th2 differentiation

**Fig 6:**
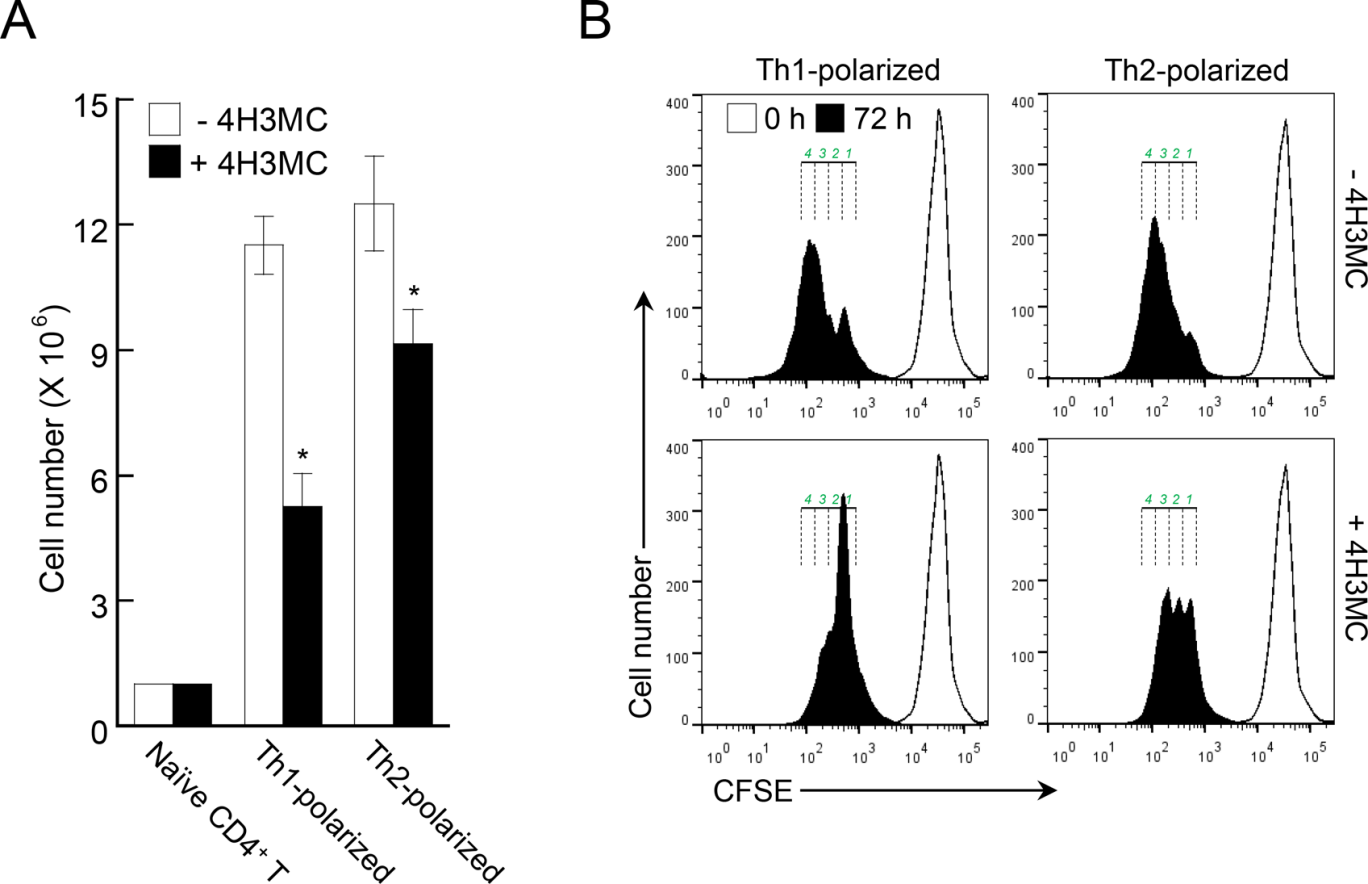
4H3MC attenuates T cell proliferation during T cell differentiation. (A) CD4^+^ T cells from LNs and spleens were differentiated into Th1- or Th2-polarized cells, as described in Materials and Methods, in the absence or presence of 4H3MC (10 μM) for 5 days. On Day 5, cells were harvested and total cell numbers measured. (B) CD4^+^ T cells from LNs and spleens were labeled with CFSE (10 μM) for 30 min and differentiated in the absence or presence of 4H3MC. After 72 h of culture, cells were harvested and the CFSE intensity measured by flow cytometry. 0 h indicates naïve CD4^+^ T cells. The numbers above 72 h population indicates the number of cell division. Data are representative of three independent experiments. Data are expressed as the mean ± SD from three independent experiments. *P < 0.05, *versus* the cell not treated with 4H3MC.

While studying the effects of 4H3MC on T cell differentiation *in vitro*, we noticed an apparent reduction in the number of cells after T cell differentiation in the presence of 4H3MC compared with that in the controls. Therefore, we counted the number of cells that differentiated into Th1 and Th2 cells. Indeed, 4H3MC reduced the number of both Th1 and Th2 cells (Fig 6A). To examine whether 4H3MC suppresses the proliferation of T cells during differentiation, we stained CD4^+^ T cells with a fluorescent dye (CFSE) and cultivated them with specific cytokines and antibodies (to induce Th1- or Th2-polarization) in the absence or presence of 4H3MC. At 72 h post-incubation, the CFSE-labeled Th1 and Th2 cells were subjected to flow cytometry. As shown in Fig 6B, the proliferation of both Th1- and Th2-polarized CD4^+^ T cells was inhibited in the presence of 4H3MC, suggesting that 4H3MC inhibits T cell proliferation but does not induce cell death during differentiation into effector cells.

### 4H3MC reduces the production of pro-inflammatory cytokines and blocks PKC activation in human keratinocytes

**Fig 7:**
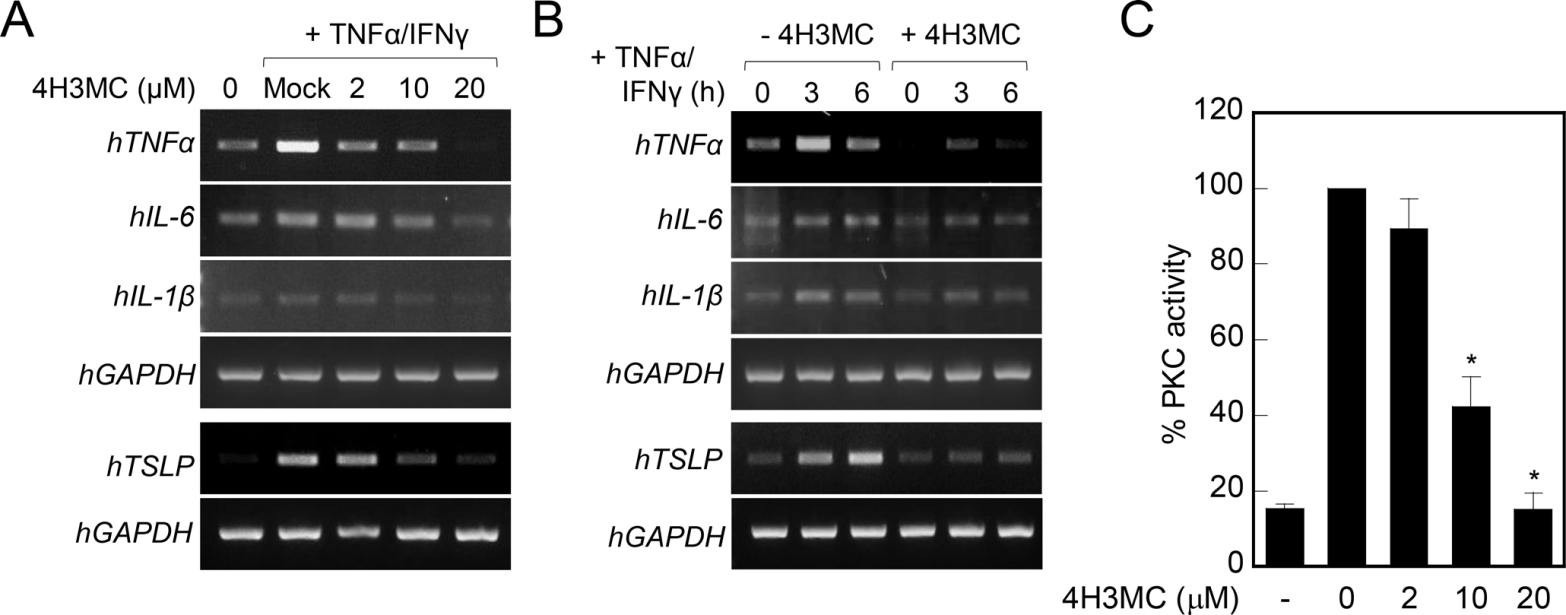
4H3MC reduces both the production of pro-inflammatory cytokines and PKC activity in keratinocytes. (A) HaCaT (1 × 10^6^) cells were pretreated with 4H3MC (0–20 μM) for 30 min and activated with TNF-α (10 ng/mL) and IFN-γ (10 ng/mL) for 3 hr and then harvested mRNA from the cells. The expression of mRNA for pro-inflammatory cytokines (TNF-α, IL-1β, IL-6 and TSLP) was measured by conventional RT-PCR. (B) HaCaT (1 × 10^6^) cells were pretreated with 4H3MC (10 μM) for 30 min and activated with TNF-α (10 ng/mL) and IFN-γ (10 ng/mL) for the indicated times (0–6 h) and then harvested mRNA from the cells. Expression of mRNA for pro-inflammatory cytokines (TNF-α, IL-1β, IL-6 and TSLP) was measured by conventional RT-PCR. Data are representative of three independent experiments. (C) HaCaT cells (1 × 10^6^) were lysed and the cytosolic fraction was incubated with various concentrations of 4H3MC (0, 2, 10, and 20 μM) for 30 min. The cell lysate was then treated with PMA (100 nM) for 3 min and 2.5 μg protein from the cytosolic fraction was used to measure PKC activity. Dara are expressed as the mean ± SD from three independent experiments. *P < 0.05, *versus* the mock-treated cells (A), or *versus* treatment with 4H3MC (B) and (C).

As T cell activation and migration in AD are controlled by activated keratinocytes [17], we next examined whether 4H3MC also affects keratinocyte activation. To this end, we looked for changes in the production of pro-inflammatory cytokines by human keratinocyte HaCaT cells stimulated by TNF-α and IFN-γ. Activated keratinocytes produced TNF-α, IL-6, IL-1β, and TSLP; however, 4H3MC led to a dose-dependent reduction in the levels of all of these cytokines (Fig 7A). The reduction in cytokine production by 4H3MC was observed at 3 h post-keratinocyte activation (Fig 7B). Our previous study demonstrated that 4H3MC acts as a PKC inhibitor in T cells [12]. PKC activity is crucial for a Th2-dominated immune response during the acute phase of AD [18]; therefore, we next examined whether 4H3MC regulates PKC activity in keratinocytes. HaCaT cells were lysed and the lysates were treated with PMA. The resultant proteins were then analyzed for PKC activity. As in T cells, 4H3MC led to a dose-dependent reduction in PKC activity in keratinocytes (Fig 7C). Taken together, these results suggest that 4H3MC inhibits not only T cells but also keratinocytes by suppressing PKC activation.

## Discussion

Here, we showed that oral administration of 4H3MC effectively ameliorates the symptoms of AD *in vivo* and identified the probable underlying mechanism.

Many studies indicate that the method used to deliver of a compound to target tissues/cells is important when studying diseases in animal models. Two routes of administration are widely used in AD models: topical and oral. Topical administration results in relatively rapid action of the compound, albeit at a limited site; however, the disadvantage is that it is difficult to control the dosage. Oral administration is more convenient for AD animal studies [19] and for patients, particularly children or difficult patients. The dose is also easier to control. This is an important consideration due to concerns related to side-effects at unintended sites. Many patients with moderate to severe AD require systemic treatment for long periods [20] because they have significant systemic symptoms not limited to the skin. For this reason, oral administration of an effective compound with no side-effects is the preferred option for treating chronic AD in the long-term. Oral administration of 4H3MC to AD mice ameliorated systemic AD symptoms: the size of enlarged dLNs and spleen was reduced, cytokine production in dLNs was reduced, and levels of IgE in the serum were reduced. However, long-term oral administration of 4H3MC (a daily dose for 20 days) had no significant side-effects in terms of the systemic immune response. Treatment with 4H3MC alone did not alter the basal levels of IgE in the serum (Fig 1D). In addition, the weight of mice receiving 4H3MC alone was comparable with that of normal mice, as was the size of the dLNs and spleens; also, the ability of CD4^+^ T cells in the dLNs to produce cytokines was not impaired (Fig 4), indicating that oral administration of 4H3MC has no unintended side-effects with respect to immune organs.

The weight and size of the dLNs, as well as cytokine production by dLN-derived T cells, was higher in AD mice than in control mice. This increase is attributable to the recruitment of immune cells (T cells, DCs, and B cells). Treatment with 4H3MC, however, suppressed T cell and DC population, but had no significant effect on B cell population. These data imply that 4H3MC regulates T cell trafficking as well as T cell proliferation. As 4H3MC inhibits PKC activation in T cells [12], it is conceivable that 4H3MC might limit T cell trafficking by regulating the PKC signaling pathways that drive cell migration. Given that PKC is ubiquitously expressed in immune and nonimmune cells, and that it governs diverse cellular processes including cell migration and gene expression [21], it is conceivable that 4H3MC might regulate the migration of DCs, mast cells, and keratinocytes, and also regulate cytokine production by keratinocytes, thereby activating DCs to prime naïve T cells to differentiate into Th2 cells during AD development. Indeed, 4H3MC suppressed the expression of TSLP as well as TNF-α, IL-6, and IL-1β. These findings suggest that 4H3MC alleviates the development of AD by inhibiting PKC activation in T cells and keratinocytes.
